# Correction: Integrated all-solid-state sulfite sensors modified with two different ion-to-electron transducers: rapid assessment of sulfite in beverages

**DOI:** 10.1039/d1ra90110a

**Published:** 2021-05-13

**Authors:** Hisham S. M. Abd-Rabboh, Abd El-Galil E. Amr, Ayman H. Kamel, Mohamed A. Al-Omar, Ahmed Y. A. Sayed

**Affiliations:** Department of Chemistry, Faculty of Science, Ain Shams University Cairo 11566 Egypt ahkamel76@sci.asu.edu.eg +20-1000361328; Chemistry Department, Faculty of Science, King Khalid University Abha 61413 Saudi Arabia habdrabboh@kku.edu.sa; Pharmaceutical Chemistry Department, Drug Exploration & Development Chair (DEDC), College of Pharmacy, King Saud University Riyadh 11451 Saudi Arabia malomar1@ksu.edu.sa ahmedyahia009@gmail.com aamr@ksu.edu.sa +966-565-148-750; Applied Organic Chemistry Department, National Research Center Dokki 12622 Giza Egypt

## Abstract

Correction for ‘Integrated all-solid-state sulfite sensors modified with two different ion-to-electron transducers: rapid assessment of sulfite in beverages’ by Hisham S. M. Abd-Rabboh *et al.*, *RSC Adv.*, 2021, **11**, 3783–3791, DOI: 10.1039/D0RA09903A.

The authors regret that the affiliations for one of the co-authors (Mohamed A. Al-Omar) were incorrectly shown in the original article. The correct affiliations are given here.

The Royal Society of Chemistry apologises for these errors and any consequent inconvenience to authors and readers.

## Supplementary Material

